# South-West Orthopaedic Club

**Published:** 1971-10

**Authors:** 


### South-West Orthopaedic Club

A Meeting of the Club was held at the Hereford General and County Hospitals on the 22nd May, 1971.

**A Comparison of Silastic Prostheses (Yeoman design) with Excision Arthroplasty in the Metacarpo-phalangeal Joints of the Hand** — Mr. I. M. Pinder (Bath) described a new operative technique for the insertion of a silastic prosthesis. A direct comparison was made of metacarpo-phalangeal replacement in rheumatoid arthritis with excision arthroplasty. Thirty-two replacement arthroplasties had been carried out in fourteen patients, and fifteen excision arthroplasties in twelve of these patients. The main indication for surgery was considered to be pain with evidence of bone destruction. A good cosmetic result was considered to be a bonus. The silastic prosthesis acts as a spacing device. Patients who had had both replacement and excision arthroplasties were overwhelmingly in favour of the spacing device, because of a greater effective range of movement and minimal loss of finger length.

**Anticoagulants in Hip Joint Surgery**—Dr. H. J. de Mol van Oterloo (The Hague) reported an investigation into the prophylactic use of anticoagulant therapy in total hip replacement surgery. The results indicated that the use of anticoagulants were of value in reducing the incidence of thrombo-embolic complications and that, provided the dosage was carefully controlled, it was possible to avoid post-operative wound complications.

**Quadriceps-plasty**—Mr. C. C. Jeffrey (Exeter), Mr. A. H. C. Ratliff (Bristol) and Mr. F. Brian Thomas (Hereford) gave an account of their experiences with the use of the Thompson Quadriceps-plasty. All were agreed that this was a valuable procedure.

**Analysis of a Decade of Hereford Hip Fractures**—Mr. P. V. Seal (Hereford) reported on a ten year retrospective study being carried out at Hereford on 817 patients with subcapital or inter-trochanteric fractures of the femoral neck. The results of this investigation emphasised particular problems in terms of the poor prognosis and the demands made upon the hospital and Social Services. For example, there was an overall hospital mortality of 16% and a one year mortality of 25%. The hospital service had to devote continuously 30% of its total adult beds to patients with this injury. Of those patients leaving hospital only 34% returned home, the remainder going to Cottage Hospitals or Geriatric Hospitals.

The study had also shown that hospital mortality was related closely to the physical condition of the patient and not to any operative delay and, therefore, it was considered advisable to be prepared to delay operation when indicated if the physical condition of the patient could thereby be improved.

**Subluxation in Osteoarthritis of the Hip Treated by Pauwel Osteotomy**—Mr. Max Harrison (Birmingham) reported on a small series of twelve patients who had had varus osteotomies for painful subluxating osteoarthritic hip joints. The results have been good in terms of relief of pain and retention of hip movements, and it was his impression that this operation was more valuable in this type of case than a displacement osteotomy. However, he stressed that there must be sufficient abduction possible to locate the hip.

**Premature Fusion of Knee Joint Epiphyses** — Mr. G. C. Slee (Hereford) reported on twenty-eight patients with premature fusion of knee joint epiphyses. There had been eleven cases of tuberculosis of the hip, thirteen with severe bilateral lower limb paralysis resulting from poliomyelitis and four who had had prolonged immobilisation for congenital dislocation of the hip. All the patients had been below the age of seven years and all had had prolonged immobilisation of between one and four years. The radiological changes were the development of severe osteoporosis and cyst like changes in the metaphysis. Early fusion of the epiphyses often resulted in severe shortening of the limb, in some cases by as much as five inches, and also in the development of knee deformities.

**Some Factors Affecting Absorption from Methylmethacrylate bone cement** — Mr. R. Ling (Exeter). During operations for total hip replacement employing cement fixation, continuous recording of the arterial blood pressure using sensitive equipment had shown blood pressure falls following thirty-five out of thirty-seven insertions of cement. The falls averaged 7% after insertion into the acetabulum and 11% after insertion into the femur. In spite of this, it was not considered by any means certain at the present time that these blood pressure falls were a direct consequence of absorption from the cement surface. Blood pressure changes of similar order were often found during other phases of the operation, particularly whilst rotating cutting tools were being used. Experimental work involving direct injection of monomer was not considered to be relevant to the clinical use of cement, primarily because of the activation reaction occurring during the preparation of cement mixes and its possible consequences. A consideration of the theoretical parameters affecting absorption from the cement surface in vivo compared with what is known of the concentrations of monomer actually occurring in practice lead to the suggestion that the local toxic action of the cement mix was responsible for creating a barrier to the absorption of toxic materials into the systemic circulation.

